# Factors Affecting Patients with Concurrent Deep Neck Infection and Lemierre’s Syndrome

**DOI:** 10.3390/diagnostics12040928

**Published:** 2022-04-08

**Authors:** Shih-Lung Chen, Shy-Chyi Chin, Yu-Chien Wang, Chia-Ying Ho

**Affiliations:** 1 Department of Otorhinolaryngology & Head and Neck Surgery, Chang Gung Memorial Hospital, Linkou 333, Taiwan; m7054@cgmh.org.tw; 2 School of Medicine, Chang Gung University, Taoyuan 333, Taiwan; b25chin@gmail.com (S.-C.C.); chiayingho23@gmail.com (C.-Y.H.); 3 Department of Medical Imaging and Intervention, Chang Gung Memorial Hospital, Linkou 333, Taiwan; 4 Department of Otorhinolaryngology & Head and Neck Surgery, New Taipei Municipal Tucheng Hospital, New Taipei City 236, Taiwan; 5 Division of Chinese Internal Medicine, Center for Traditional Chinese Medicine, Chang Gung Memorial Hospital, Taoyuan 333, Taiwan

**Keywords:** concurrent, carotid space, deep neck infection, Lemierre’s syndrome, posterior cervical space

## Abstract

Deep neck infection (DNI) is a severe disease affecting the deep neck spaces, and is associated with an increased risk of airway obstruction. Lemierre’s syndrome (LS) refers to septic thrombophlebitis of the internal jugular vein after pharyngeal infection, and is linked with high morbidity and mortality. Both diseases begin with an oropharyngeal infection, and concurrence is possible. However, no studies have examined the risk factors associated with co-existence of LS and DNI. Accordingly, this study examined a patient population to investigate the risk factors associated with concurrent DNI and LS. We examined data from a total of 592 patients with DNI who were hospitalized between May 2016 and January 2022. Among these patients, 14 had concurrent DNI and LS. The relevant clinical variables were assessed. In a univariate analysis, C-reactive protein (odds ratio (OR) = 1.004, 95% CI: 1.000–1.009, *p* = 0.045), involvement of multiple spaces (OR = 23.12, 95% CI: 3.003–178.7, *p* = 0.002), involvement of the carotid space (OR = 179.6, 95% CI: 22.90–1409, *p* < 0.001), involvement of the posterior cervical space (OR = 42.60, 95% CI: 12.45–145.6, *p* < 0.001) and *Fusobacterium necrophorum* (*F. necrophorum*, OR = 288.0, 95% CI: 50.58–1639, *p* < 0.001) were significant risk factors for concurrent DNI and LS. In a multivariate analysis, involvement of the carotid space (OR = 94.37, 95% CI: 9.578–929.9, *p* < 0.001), that of the posterior cervical space (OR = 24.99, 95% CI: 2.888–216.3, *p* = 0.003), and *F. necrophorum* (OR = 156.6, 95% CI: 7.072–3469, *p* = 0.001) were significant independent risk factors for concurrent LS in patients with DNI. The length of hospitalization in patients with concurrent LS and DNI (27.57 ± 14.94 days) was significantly longer than that in patients with DNI alone (10.01 ± 8.26 days; *p* < 0.001), and the only pathogen found in significantly different levels between the two groups was *F. necrophorum* (*p* < 0.001). Involvement of the carotid space, that of the posterior cervical space and *F. necrophorum* were independent risk factors for the concurrence of DNI and LS. Patients with concurrent LS and DNI had longer hospitalization periods than patients with DNI alone. Furthermore, *F. necrophorum* was the only pathogen found in significantly different levels in DNI patients with versus those without LS.

## 1. Introduction

Deep neck infection (DNI) is a life-threatening bacterial infection of the spaces formed by the deep cervical fascia [1]. It can lead to airway obstruction and cause severe morbidity, including severe cervical necrotizing fasciitis, severe sepsis, esophageal perforation, descending necrotizing mediastinitis, and even Lemierre’s syndrome (LS) [2,3,4,5,6,7,8].

LS is characterized by thrombophlebitis of the internal jugular vein and evidence of disseminated infection, especially septic pulmonary emboli after a recent oropharyngeal bacterial infection [9,10]. The most common pathogen associated with LS is *F. necrophorum* [11,12]. *F. necrophorum* is a gram-negative, obligate anaerobic, non-spore-forming and non-motile rod as the partial normal flora within the oral cavity, gastrointestinal tract as well as female genital tract [13].

Clinically, DNI is first suspected in patients with swelling of the neck, local heat, redness, dysphagia, and shortness of breath, while LS manifests as acute pharyngitis, dental pain, high fever, unilateral neck pain with tenderness, dyspnea, and chest pain. In assessments of patients with potential DNI, clinicians generally order many different types of imaging. Through this process, concurrent thrombophlebitis of the internal jugular vein is sometimes accidentally detected [12]. Previous studies have indicated that some patients present with concurrent DNI and LS [11,14,15]. Therapeutic management of these patients is complicated, as both diseases are potentially lethal.

Despite the associated treatment challenges, no previous studies have examined the risk factors associated with the co-existence of DNI and LS. Accordingly, the goal of this study was to investigate the risk factors and prognostic variables associated with concurrent DNI and LS.

## 2. Materials and Methods

**Figure 1:**
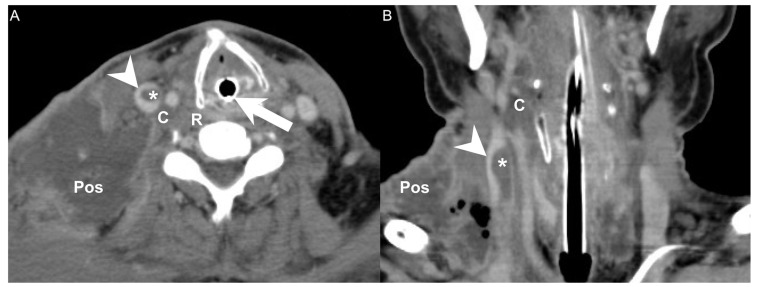
(**A**) CT axial view and (**B**) coronal view of a patient with concurrent DNI and LS. Arrowhead: internal jugular vein; Asterisk: thrombosis; Arrow: endotracheal tube; C: carotid space; Pos: posterior cervical space; R: retropharyngeal space.

**Figure 2:**
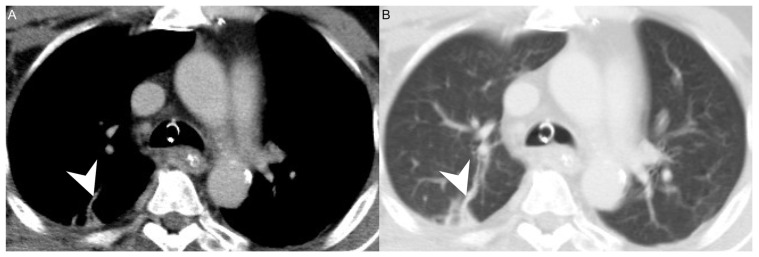
(**A**) CT axial view and (**B**) lung window of a patient with concurrent DNI and LS. Arrowhead: septic emboli.

We retrospectively reviewed the medical records of 592 patients diagnosed with DNI who were admitted to Chang Gung Memorial Hospital in Linkou, a tertiary medical center in Taiwan, between May 2016 and January 2022. Diagnoses were performed according to clinical presentation, ultrasonography (US) [16] and computed tomography (CT) of the head and neck (Figure 1A,B), and CT of the chest (Figure 2A,B) [11]. Treatment included antibiotics, US-guided needle drainage, open surgical incision, drainage of abscesses, and pleural drainage. The empirical antibiotics used were ceftriaxone (1 gm q12h) and metronidazole (500 mg q8h), based on previous reports, to address aerobic and anaerobic bacteria before the culture results were available [11,17,18,19].

### 2.1. Exclusion Criteria

Patients with severe cardiopulmonary diseases, previous head or neck tumor surgery, previous radiotherapy of the head and neck region, and a history of mis-swallowing of a foreign body were excluded from analysis. A total of 592 patients with DNI were included in the study, among whom 14 had concurrent LS.

### 2.2. Data Collection

To investigate the risk factors associated with concurrent DNI and LS, we collected data on the patients’ gender, age, C-reactive protein (CRP) level, blood sugar level, diabetes mellitus (DM) status, number of spaces affected by DNI, deep neck space involvement, presence of mediastinitis, length of hospital stay, intubation, tracheostomy, performance of open surgery for incision and drainage (I&D), sites of metastatic septic emboli, and species of pathogens involved.

### 2.3. Statistical Analysis

All data were analyzed using MedCalc software (ver. 18.6; MedCalc, Ostend, Belgium). As the Kolmogorov–Smirnov test showed that the data were not normally distributed, the Chi-square test was used for categorical variables, the Mann–Whitney *U* test was used for continuous variables, and logistic regression was used for univariate and multivariate analyses. A multivariate forward stepwise selection procedure was implemented, and all of the variables included in the univariate analysis were entered into the final multivariate model. In all analyses, *p* < 0.05 was taken to indicate statistical significance.

## 3. Results

Demographic and clinical data are shown in Table 1. We analysed data from a total of 592 patients with DNI, including 365 men (61.65%) and 227 women (38.35%) with a mean age of 52.06 ± 18.55 years. With regard to laboratory data, the mean CRP level was 150.37 ± 106.35 mg/L and the mean blood sugar level was 143.30 ± 71.61 mg/dL. A total of 250 (42.29%) patients had DM.

A total of 196 (33.10%) patients had DNI affecting a single deep neck space, 175 (29.56%) had infections in two spaces, and 221 (37.34%) had infections in more than three spaces. Of those with deep neck space involvement, the parapharyngeal space was affected in 336 (56.75%) patients, the submandibular space in 268 (45.27%), the retropharyngeal space in 233 (39.35%), the masticator space in 137 (23.14%), the parotid space in 96 (16.21%), the anterior cervical space in 56 (9.45%), the visceral space in 52 (8.78%), the carotid space in 52 (8.78%), the perivertebral space in 32 (5.40%), and the posterior cervical space in 16 (2.70%). Mediastinitis was found in 82 (13.85%) patients. The mean length of hospital stay was 10.41 ± 8.87 days. Intubation was performed in 280 (47.29%) patients, and tracheostomies were performed in 103 (17.39%) patients. A total of 276 (46.62%) DNI patients underwent open surgery for I&D.

Table 1 lists the pathogens cultured from the patients. The overall rate of specific pathogen non-growth was 18.41% (109/592). Concurrent LS was found in 14 (2.36%) patients. The sites of metastatic septic emboli included the lung (1.35%, 8/592) and brain (0.33%, 2/592).

Table 2 shows the results of the univariate analysis of variables for the 592 patients with DNI. The results showed that CRP (odds ratio (OR) = 1.004, 95% CI: 1.000–1.009, *p* = 0.045), involvement of multiple spaces (OR = 23.12, 95% CI: 3.003–178.7, *p* = 0.002), involvement of the carotid space (OR = 179.6, 95% CI: 22.90–1409, *p* < 0.001), and involvement of the posterior cervical space (OR = 42.60, 95% CI: 12.45–145.6, *p* < 0.001) were significant risk factors for concurrent DNI and LS. In Table 2, all factors were entered into a forward stepwise multivariate logistic regression model. Involvement of the carotid space (OR = 94.37, 95% CI: 9.578–929.9, *p* < 0.001), that of the posterior cervical space (OR = 24.99, 95% CI: 2.888–216.3, *p* = 0.003), and *F. necrophorum* (OR = 156.6, 95% CI: 7.072–3469, *p* = 0.001) were significant independent risk factors for concurrent LS in patients with DNI.

Table 3 shows the length of hospitalization and management of 14 patients with concurrent DNI and LS compared with those of 578 patients with DNI alone. Significant differences between the two groups were detected in terms of length of hospitalization (*p* < 0.001). The length of hospitalization in patients with concurrent LS and DNI (27.57 ± 14.94 days) was significantly longer than that in patients with DNI alone (10.01 ± 8.26 days).

Table 4 displays the pathogens found in 14 patients with concurrent DNI and LS compared with those found in 578 patients with DNI alone. The only pathogen found in significantly different levels between the two groups was *F. necrophorum* (*p* < 0.001). In addition, there were no significant differences in the absence of growth of specific pathogens from blood cultures between patients with concurrent LS and DNI (21.42%, 3/14) and patients with DNI alone (18.33%, 106/578; *p* = 0.774).

## 4. Discussion

DNI can lead to severe and life-threatening complications, while LS is characterized by imaging evidence of internal jugular vein thrombosis, clinical oropharyngeal infection, and the isolation of anaerobic pathogens, mainly *F. necrophorum* [20]. The management of concurrent DNI and LS requires airway protection, including intubation and tracheostomy [21]. Further, broad-spectrum intravenous antibiotics, potential anticoagulants, and drainage of abscesses are frequently necessary [14]. In the present study, we found that involvement of the carotid space and posterior cervical space were independent risk factors associated with concurrent DNI and LS. In addition, patients with concurrent LS and DNI had a longer hospitalization period, as compared to patients with DNI alone. Furthermore, *F. necrophorum* was the only pathogen found in significantly different levels in DNI patients with versus those without LS.

As both DNI and LS can lead to life-threatening medical situations, early diagnosis is essential. DNI can be diagnosed via flexible fiberoscopy and CT. For LS, Doppler US can be used to visualize an echogenic region within a dilated internal jugular vein or a complex mass of cystic and solid components [22]. CT findings indicative of internal jugular vein thrombus include vessel expansion, surrounding inflammatory change, and edema [14,23]. MRI is not routinely used for primary diagnoses of internal jugular vein thrombosis because it is often expensive and inconvenient in acute settings [24].

As shown in Table 2, a higher CRP level and the presence of multiple infected spaces were risk factors for concurrent DNI and LS in our univariate analysis. CRP is an acute inflammatory protein that is released in response to pro-inflammatory cytokines during infectious processes [25]. In this study, the DNI patients with concurrent LS had an average mean CRP level of 207.72 ± 19.63 mg/L, which was about 1.5-fold greater than that in the patients without concurrent LS (CRP level: 148.98 ± 107.21 mg/L, *p =* 0.045).

Consistent with our research, previous studies have indicated that patients with DNI and a CRP level >100 mg/L have longer hospitalization periods [26]. However, the CRP levels in our multivariate analysis did not show variations that reached statistical significance. Indeed, higher CRP levels are representative of greater infection severity, and a previous study found that higher CRP was associated with an enhanced risk of complications in DNI patients [8]. However, if severe abscesses did not occur at a critical site, a high CRP level did not necessarily predict the concurrence of DNI and LS.

The involvement of multiple spaces (≥3 spaces) was another risk factor revealed by our univariate analysis. Previous research has indicated that DNI is more advanced and severe when multiple spaces are affected [27]. However, in our dataset, the involvement of multiple spaces did not always mean that the internal jugular vein was affected. Thus, the involvement of multiple spaces was not an independent risk factor in the multivariate analysis.

In this study, involvement of the carotid space and of the posterior cervical space were both independent risk factors for concurrent DNI and LS in the univariate and multivariate analyses [23]. The carotid space is a space defined by the carotid sheath, which comprises the superficial, middle, and deep layers of the deep cervical fascia. Extending from the jugular foramen at the skull base to the aortic arch, the carotid space contains the internal jugular vein, internal carotid artery, cranial nerves, sympathetic plexus, and deep cervical lymph nodes [24,28]. The posterior cervical space, extending from the skull base to the clavicle, refers to the posterior and lateral aspects of the neck and constitutes a major portion of the posterior triangle of the neck [29]. The posterior cervical space is anteromedially separated from the carotid space by the carotid sheath. The main contents of the posterior cervical space are fat, spinal accessory nerves, and the spinal accessory lymph node chain [30,31]. Thrombosis of the internal jugular veins can have many causes, including head and neck infections such as LS, extrinsic compression, a chronic indwelling catheter, malignancy, and hypercoagulability syndromes [24]. Because the internal jugular vein is adjacent to the posterior cervical space and the carotid space (Figure 1), simultaneous infection of both regions may increase the chance of concurrent DNI and LS.

Airway protection, antibiotics, and repeated surgical draining of neck abscesses are essential for DNI treatment, while the treatment modalities for LS include antibiotics, anticoagulants, and surgical treatments such as intercostal drainage of a pneumothorax [12,13]. The efficacy of anticoagulants in treating LS remains controversial [11]. Some authors have concluded that there is no evidence supporting a beneficial effect of anticoagulant therapy for LS patients [32], while others favored initial anticoagulant therapy using heparin followed by warfarin [33]. A few authors have recommended anticoagulants only if thrombosis extends into the cerebral sinuses or if there has been no improvement in symptoms with antibiotic therapy alone [34,35,36]. Thus, it is difficult to establish an evidence-based guideline for the management of patients with LS. Schulman et al. found that over 99% of patients with LS were treated with antibiotics, whereas only 56% received anticoagulants during or after hospitalization [10].

Of our 14 patients with concurrent LS and DNI, 11 (78.57%) were treated with anticoagulants. The other three had limited internal jugular vein thrombophlebitis, a contraindication to anticoagulation, and a heightened a risk of major bleeding, so anticoagulants were not given. In addition, jugular vein thrombosis can lead to inflammation and consequent septic thrombophlebitis, which gives rise to distant emboli that usually migrate to pulmonary vessels (Figure 2) [37]. As a consequence, the most frequently involved sites of septic metastases are the lungs. In some patients, septic pulmonary lesions require drainage or pleural decortication [10]. Other sites involved in septic metastasis and abscess formation are the muscles, soft tissue, liver, spleen, kidneys, and central nervous system [37,38,39].

For patients with concurrent DNI and LS, we believe that protection of the airway, drainage of deep neck space abscess, and control of infection via antibiotics are necessary actions. Further, in LS patients with no contraindications, the administration of anticoagulants can reduce the chance of complications caused by septic emboli. Given the potential severity of concurrent DNI and LS and the associated risk of major complications, we suggest that these patients be treated in the intensive care unit with a multidisciplinary team including an infectious disease specialist, respirologist, otorhinolaryngologist, anesthesiologist, and cardiovascular surgeon [40,41].

As shown in Table 4, *F. necrophorum*, a gram-negative anaerobe, was the only pathogen found in significantly different levels in DNI patients with and without LS. Previous studies have indicated that this pathogen is the most frequent culprit of LS [10,11,14,42]. Ramirez et al. speculated that the recent increase in the number of serious infections caused by *F. necrophorum* could be attributed to regional alterations in antibiotic usage patterns [43]. *F. necrophorum* is usually susceptible to penicillin, clindamycin, metronidazole, and chloramphenicol, but there is a varied response to the 2nd and 3rd generations of cephalosporins [14,44]. The suggested treatment for *F. necrophorum* is a combination of beta-lactam antibiotics (penicillin/cephalosporin) with anaerobic antimicrobial agents such as metronidazole, clindamycin and tazocin [45], while macrolides, fluoroquinolones, tetracyclines, and aminoglycosides should be avoided [40,41]. Kim et al. successfully used intravenous piperacillin + tazobactam with subsequent oral amoxicillin + clavulanic acid to manage sternoclavicular septic arthritis caused by resistant *F. necrophorum*. Thus, the options for beta-lactam resistant *F. necrophorum* infection include the combination of piperacillin + tazobactam as well as amoxicillin + clavulanic acid [46]. The culture of *F. necrophorum* takes 6 to 8 days to grow, which results in delayed choices regarding adequate antibiotics treatment [47,48]. Thus, patients with concurrent DNI and LS should initially be treated with empirical, broad-spectrum, intravenous antibiotics, and these should then be adjusted according to the results of the wound culture.

In Table 4, *Streptococcus constellatus* (*S. constellatus*, 20.27%, 120/592) was the most cultivated pathogen in this research. This pathogen belongs to the *Streptococcus milleri* group, a subgroup of viridans streptococci that consists of three streptococcal species, which are *S. constellatus*, *S. anginosus* and *S. intermedius* [49,50]. These organisms are commonly found on the mucous membrane of the oral cavity as well as oral pharynx. They can become pathogenic to cause severe suppurative infection after mucosal disruption even though they are commensal organisms. They also form abscesses and have local aggressive extension to surrounding tissues such as deep neck spaces [51].

The rate of specific pathogen non-growth in this research was 18.41% (109/592). However, blood culture is not a highly sensitive method for identifying pathogens, and the negative culture results may be due to the use of antibiotics prior to admission or intravenous antibiotic treatment before surgical drainage [17].

## 5. Limitations

This study had several limitations. First, the sizes of the two groups differed. The concurrent LS and DNI group (n = 14) was only 2% compared to the DNI alone group (n = 578). Second, the retrospective nature of the study resulted in a certain attrition rate. In addition, most of the patients were male. A better designed case-control study in the future would conquer the inherited flaws and bias that existed in this retrospective study.

## 6. Conclusions

The carotid space, posterior cervical space and *F. necrophorum* were independent risk factors for the concurrence of DNI and LS. Patients with concurrent LS and DNI had a longer hospitalization period compared with patients with DNI alone. Furthermore, *F. necrophorum* was the only pathogen found in significantly different numbers between DNI patients with versus those without LS.

## Figures and Tables

**Table 1:** Clinical characteristics of 592 patients with DNI.

Characteristics	N (%)
Gender	592 (100.00)
Male	365 (61.65)
Female	227 (38.35)
Age, years (SD)	52.06 ± 18.55
CRP, mg/L (SD)	150.37 ± 106.35
Blood sugar, mg/dL (SD)	143.30 ± 71.61
Diabetes mellitus	250 (42.29)
Number of deep neck space involvement	
Single space	196 (33.10)
Double spaces	175 (29.56)
Multiple spaces, ≥3	221 (37.34)
Deep neck space involvement	
Parapharyngeal space	336 (56.75)
Submandibular space	268 (45.27)
Retropharyngeal space	233 (39.35)
Masticator space	137 (23.14)
Parotid space	96 (16.21)
Anterior cervical space	56 (9.45)
Visceral space	52 (8.78)
Carotid space	52 (8.78)
Perivertebral space	32 (5.40)
Posterior cervical space	16 (2.70)
Mediastinitis	82 (13.85)
Length of hospital stay, days (SD)	10.41 ± 8.87
Intubation	280 (47.29)
Tracheostomy	103 (17.39)
Incision and drainage open surgery	276 (46.62)
Pathogens	
*Streptococcus constellatus*	120 (20.27)
*Prevotella intermedia*	73 (12.33)
*Streptococcus anginosus*	65 (10.97)
*Klebsiella pneumoniae*	64 (10.81)
*Parvimonas micra*	63 (10.64)
*Prevotella buccae*	59 (9.96)
*Staphylococcus aureus*	35 (5.91)
*Streptococcus salivarius*	23 (3.88)
*Streptococcus pneumoniae*	22 (3.71)
*Staphylococcus epidemidis*	22 (3.71)
*Serratia marcescens*	22 (3.71)
*Streptococcus oralis*	20 (3.37)
*Gemella morbillorum*	19 (3.20)
*Eikenella corrodens*	18 (3.04)
*Salmonella enterica*	16 (2.70)
*Slackia exigua*	11 (1.85)
*Rothia amarae*	10 (1.68)
*Pseudomonas aeruginosa*	9 (1.52)
*Stenotrophomonas maltophilia*	9 (1.52)
*Fusobacterium necrophorum*	9 (1.52)
*Burkholderia gladioli*	8 (1.35)
No growth	109 (18.41)
LS	14 (2.36)
Sites of metastatic septic emboli	
Lung	8 (1.35)
Brain	2 (0.33)

DNI = deep neck infection; N = numbers; SD = standard deviation; CRP = C-reactive protein (normal range <5 mg/L); Blood sugar (normal range: 70–100 mg/dL); LS = Lemierre’s syndrome.

**Table 2:** Univariate and multivariate analyses of data from 14 patients with concurrent DNI and LS compared with those from 578 patients with DNI alone.

Variable	LS		Univariate Analysis			Multivariate Analysis		
	Yes	No	OR	95% CI	*p* Value	OR	95% CI	*p* Value
Gender	14	578			0.837			
Male	9	356	1.122	0.330–3.022				
Female	5	222	1.000					
Age, years					0.503			
>60	4	216	0.670	0.207–2.163				
≤60	10	362	1.000					
CRP, mg/L (SD)	207.72 ± 19.63	148.98 ± 107.21	1.004	1.000–1.009	0.045 *	-	-	-
Blood sugar, mg/dL (SD)	150.35 ± 20.43	143.13 ± 72.40	1.001	0.994–1.008	0.709			
Diabetes mellitus					0.284			
Yes	4	246	0.539	0.167–1.741				
No	10	332	1.000					
Multiple spaces, ≥3					0.002 *	-	-	-
Yes	13	208	23.12	3.003–178.7				
No	1	370	1.000					
Parapharyngeal space					0.270			
Yes	10	326	1.934	0.309–3.225				
No	4	525	1.000					
Submandibular space					0.370			
Yes	8	260	1.630	0.558–4.759				
No	6	318	1.000					
Retropharyngeal space					0.063			
Yes	9	224	2.844	0.941–8.596				
No	5	354	1.000					
Masticator space					0.087			
Yes	6	131	2.559	0.872–7.508				
No	8	447	1.000					
Parotid space					0.055			
Yes	5	91	2.973	0.974–9.075				
No	9	478	1.000					
Anterior cervical space					0.174			
Yes	3	53	2.701	0.730–9.987				
No	11	525	1.000					
Visceral space					0.495			
Yes	2	50	1.760	0.383–8.085				
No	12	528	1.000					
Carotid space					<0.001 *			<0.001 *
Yes	13	39	179.6	22.90–1409		94.37	9.578–929.9	
No	1	539	1.000			1.000		
Perivertebral space					0.209			
Yes	2	30	3.044	0.651–14.22				
No	12	548	1.000					
Posterior cervical space					<0.001 *			0.003 *
Yes	6	10	42.60	12.45–145.6		24.99	2.888–216.3	
No	8	568	1.000			1.000		
Mediastinitis					0.119			
Yes	4	78	2.564	0.784–8.376				
No	10	500	1.000					
*Fusobacterium necrophorum*					<0.001 *			0.001 *
Yes	7	2	288.0	50.58–1639		156.6	7.072–3469	
No	7	576	1.000			1.000		

DNI = deep neck infection; LS = Lemierre’s syndrome; SD = standard deviation; OR = odds ratio; CI = confidence intervals; CRP = C-reactive protein; *, *p* < 0.05. Significant differences are shown in bold.

**Table 3:** Length of hospitalization and management of 14 patients with concurrent DNI and LS compared with those of 578 patients with DNI alone.

Characteristics	LS, N = 14 (%)	Non-LS, N = 578 (%)	*p* Value
Length of hospital stay, days (SD)	27.57 ± 14.94	10.01 ± 8.26	<0.001 *
Intubation			1.000
Yes	7 (50.00)	273 (47.23)	
No	7 (50.00)	305 (52.77)	
Tracheostomy			0.078
Yes	5 (35.71)	98 (16.95)	
No	9 (64.29)	480 (83.05)	
I&D open surgery			1.000
Yes	6 (42.85)	270 (46.71)	
No	8 (57.15)	308 (53.29)	

DNI = deep neck infection; LS = Lemierre’s syndrome; N = number; I&D = incision and drainage; *, *p* < 0.05. Significant differences are shown in bold.

**Table 4:** Pathogens detected in cultures from 14 patients with concurrent DNI and LS compared with those from 578 patients with DNI alone.

Pathogens	LS, N = 14 (%)	Non-LS, N = 578 (%)	*p* Value
*Streptococcus constellatus*	2 (14.28)	118 (20.41)	0.557
*Prevotella intermedia*	2 (14.28)	71 (12.28)	0.829
*Streptococcus anginosus*	2 (14.28)	63 (10.89)	0.700
*Klebsiella pneumoniae*	2 (14.28)	62 (10.72)	0.694
*Parvimonas micra*	0 (0.00)	63 (10.89)	0.073
*Prevotella buccae*	0 (0.00)	59 (10.20)	0.201
*Staphylococcus aureus*	2 (14.28)	33 (5.70)	0.248
*Streptococcus salivarius*	0 (0.00)	23 (3.97)	0.288
*Streptococcus pneumoniae*	1 (7.14)	21 (3.63)	0.539
*Staphylococcus epidemidis*	1 (7.14)	21 (3.63)	0.503
*Serratia marcescens*	2 (14.28)	20 (3.46)	0.100
*Streptococcus oralis*	0 (0.00)	20 (3.46)	0.322
*Gemella morbillorum*	0 (0.00)	19 (3.28)	0.335
*Eikenella corrodens*	0 (0.00)	18 (3.11)	0.349
*Salmonella enterica*	0 (0.00)	16 (2.76)	0.378
*Slackia exigua*	0 (0.00)	11 (1.90)	0.465
*Rothia amarae*	1 (7.14)	9 (1.55)	0.226
*Pseudomonas aeruginosa*	1 (7.14)	8 (1.38)	0.200
*Stenotrophomonas maltophilia*	1 (7.14)	8 (1.38)	0.200
*Fusobacterium necrophorum*	7 (50.00)	2 (0.34)	<0.001 *
*Burkholderia gladioli*	1 (7.14)	7 (1.21)	0.174
No growth	3 (21.42)	106 (18.33)	0.774

DNI = deep neck infection; LS = Lemierre’s syndrome; N = number; *, *p* < 0.05. Significant differences are shown in bold.

## Data Availability

All data generated or analyzed during this study are included in this published article. The data are available on request.

## Abbreviations

CT: Computed tomography; CRP: C-reactive protein; CI: confidence interval; DNI: Deep neck infection; DM: Diabetes mellitus; I&D: incision and drainage; LS: Lemierre’s syndrome; OR: Odds ratio; US: Ultrasonography.
